# Effect of testosterone deficiency on cholesterol metabolism in pigs fed a high-fat and high-cholesterol diet

**DOI:** 10.1186/s12944-015-0014-5

**Published:** 2015-03-07

**Authors:** Zhaowei Cai, Haitao Xi, Yongming Pan, Xiaoling Jiang, Liang Chen, Yueqin Cai, Keyan Zhu, Cheng Chen, Xiaoping Xu, Minli Chen

**Affiliations:** Laboratory Animal Research Center, Zhejiang Chinese Medical University, Hangzhou, 310053 China; Reproduction Medicine Center, The Second Affiliated Hospital of Wenzhou Medical University, Wenzhou, 325027 China; College of Animal Science, Zhejiang University, Hangzhou, 310025 China

**Keywords:** Testosterone, Atherosclerosis, Hypercholesterolemia, Miniature pigs, Gene expression

## Abstract

**Background:**

Testosterone deficiency is associated with increased serum cholesterol levels. However, how testosterone deficiency precisely affects cholesterol metabolism remains unclear. Therefore, in the current study, we examined the effect of testosterone deficiency on cholesterol metabolism and liver gene expression in pigs fed a high-fat and high-cholesterol (HFC) diet.

**Methods:**

Sexually mature male miniature pigs (6–7 months old) were randomly divided into 3 groups as follows: intact male pigs fed an HFC diet (IM + HFC), castrated male pigs fed an HFC diet (CM + HFC), and castrated pigs with testosterone replacement fed an HFC diet (CM + HFC + T). Serum testosterone levels and lipid profiles were measured, and gene expression levels associated with hepatic cholesterol metabolism were determined. Furthermore, total hepatic cholesterol contents and the activities of enzymes mediating hepatic cholesterol metabolism were measured.

**Results:**

Serum testosterone levels were significantly decreased in CM + HFC pigs, and testosterone replacement attenuated castration-induced testosterone deficiency. Castration significantly increased the serum levels of total cholesterol, low-density lipoprotein cholesterol and triglycerides, as well as hepatic lipid contents in pigs fed an HFC diet. Compared with IM + HFC and CM + HFC + T pigs, low-density lipoprotein receptor (*LDLR*) mRNA expression and protein levels were significantly decreased in the livers of CM + HFC pigs. In contrast, we found that compared with IM + HFC pigs, hepatic proprotein convertase subtilisin/kexin type 9 (*PCSK9*) mRNA and serum PCSK9 protein levels were significantly increased in CM + HFC pigs. Moreover, testosterone treatment reversed the increase in PCSK9 expression in CM + HFC pigs. However, neither castration nor testosterone replacement affected the expression of the other hepatic genes that were tested.

**Conclusions:**

This study demonstrated that castration-induced testosterone deficiency caused severe hypercholesterolemia in pigs fed an HFC diet; furthermore, these effects could be reversed by testosterone replacement therapy. Altered hepatic PCSK9 and LDLR expression, resulting in reduced LDL-cholesterol clearance, may contribute to the increased serum cholesterol levels induced by testosterone deficiency and an HFC diet. These results deepen our understanding of the underlying molecular mechanisms that mediate the effects of testosterone deficiency on cholesterol metabolism.

## Background

Aging in men causes a gradual decline in endogenous testosterone levels [1], which may have detrimental effects on health status [2]. Numerous studies have shown that low levels of endogenous testosterone are associated with an increased risk of atherosclerosis in men [3-5], and serum lipids are essential for atherosclerosis development [6,7]. Testosterone deficiency is thought to promote atherosclerosis by modulating lipid metabolism [8]. Several clinical and epidemiological studies have reported that serum testosterone levels are inversely correlated with total cholesterol and LDL cholesterol levels [9-11]. Moreover, animal studies have also demonstrated markedly increased serum cholesterol levels in testosterone-deficient male mice [12,13]. These findings indicate that testosterone serves an important role in regulating serum cholesterol metabolism. However, to date the potential molecular mechanisms whereby testosterone deficiency affects cholesterol metabolism are unclear.

The liver is a critical organ for normal cholesterol metabolism and contains numerous proteins and enzymes related to cholesterol homoeostasis. Three well-studied examples of such enzymes include 3-hydroxy-3-methylgutaryl coenzyme A reductase (HMGCR), which is the rate-limiting enzyme in cholesterol synthesis; the low-density lipoprotein receptor (LDLR), which is responsible for the removal of LDL-cholesterol from the blood; and cholesterol 7α-hydroxylase (CYP7A1), which is the main enzyme responsible for catalyzing the conversion of cholesterol into bile acids and reverse cholesterol transport [14]. Furthermore, sterol regulatory element-binding protein 2 (SREBP2) governs transcriptional activation of *LDLR* and *HMGCR*, whereas liver X receptor alpha (LXRα) regulates the transcription of *CYP7A1* [15]. To date, limited data are available regarding the effects of testosterone on the modulation of hepatic cholesterol homeostasis-related proteins. Therefore, it is unknown whether a testosterone deficiency-induced increase in serum cholesterol levels is related to changes in hepatic protein expression that are involved in cholesterol metabolism.

Here, we aimed to determine the effect of testosterone deficiency on cholesterol metabolism in pigs fed an HFC diet. In additional, we explored potential associated mechanisms by measuring the expression of genes related to hepatic cholesterol metabolism.

## Methods

### Animals and experimental procedures

**Figure 1:**
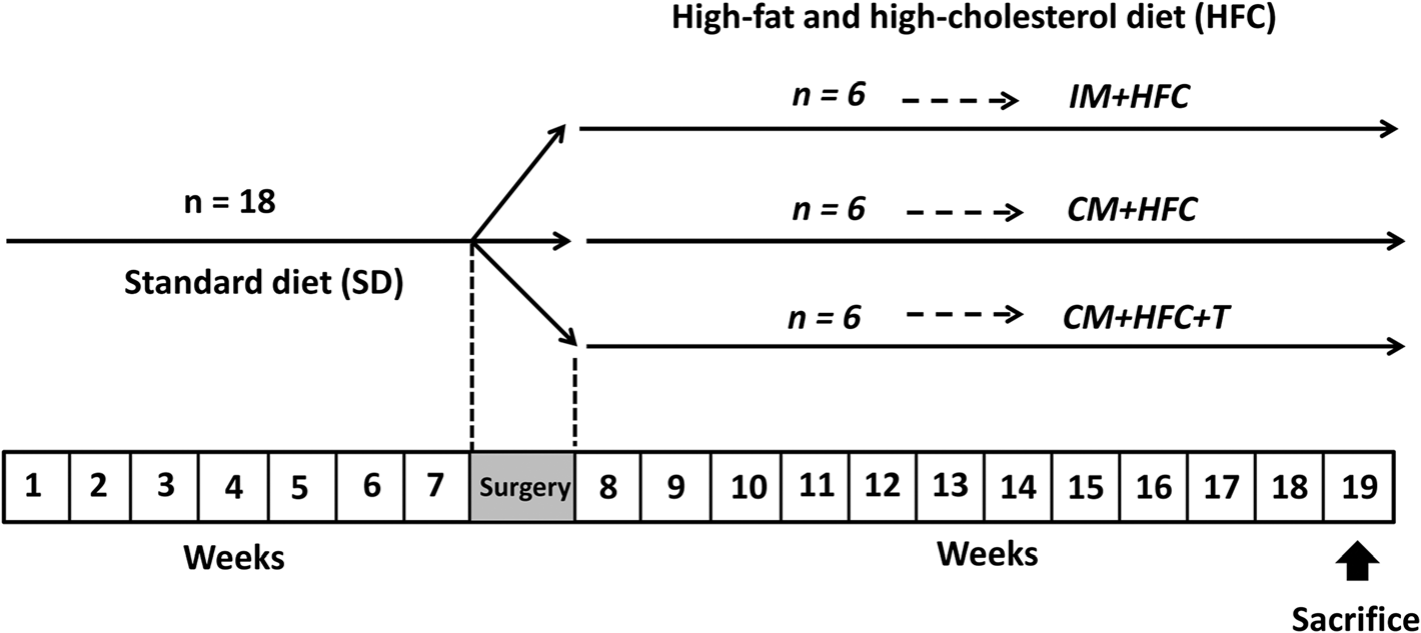
**Schematic representation of the experimental design.** Sexually mature male miniature pigs (6–7 months old) were fed a standard diet without cholesterol for 7 weeks. At week 7, the pigs were either surgically castrated or given a sham operation. Moreover, testosterone replacement therapy was given on the day of castration. Beginning at week 8, intact male pigs (IM + HFC), castrated pigs (CM + HFC), and castrated male pigs with testosterone replacement (CM + HFC + T) were fed the same high-fat and high cholesterol (HFC) diet for another 12 weeks, after which they were euthanized.

All experimental procedures used in this study were approved by the Institutional Animal Care and Use Committee of the Zhejiang Chinese Medical University (Hangzhou, China). Eighteen sexually mature male Chinese Wuzhishan (WZS) miniature pigs (6–7 months old) were obtained from the Institute of Animal Sciences, Hainan Academy of Agricultural Sciences (Haikou, China). The animals were housed in individual pens under environmental conditions with a room temperature of 22°C ± 3°C, a relative humidity of 50% ± 20% and a 12-hour light/dark cycle. The study protocol is outlined in Figure 1. The animals received a standard diet without cholesterol during a 7-week “pretreatment period” to facilitate acclimation to the environment and baseline determinations. At week 7, the pigs were either surgically castrated or given a sham operation, as described previously [16]. Testosterone was administrated weekly to castrated pigs via intramuscular injection with testosterone propionate (10 mg/kg body weight; Sigma-Aldrich, St. Louis, MO, USA) dissolved in corn oil [17]. Testosterone replacement therapy was given on the same day of castration to avoid the disruption of hormonal influences. Pigs were fed an HFC diet starting from week 8 and were divided into 3 groups (n = 6 animals/group) as follows: intact male pigs fed an HFC diet (IM + HFC), castrated male pigs fed an HFC diet (CM + HFC), and castrated pigs with testosterone replacement fed an HFC diet (CM + HFC + T). The HFC diet used in this study was comprised of 73% normal swine diet, 15% lard, 10% egg yolk power, 1.5% cholesterol, and 0.5% sodium cholate. The HFC diet was similar to an atherogenic diet, which has been shown to induce hypercholesterolemia and atherosclerosis in pigs [18,19]. Body weights were recorded weekly, and the study period was 12 weeks.

At the end of the experimental period, the animals were sacrificed by exsanguination under anesthesia. The carcasses were eviscerated as previously described [20], livers were removed and weighed, and liver weight indexes were calculated as liver weight/body weight ratios. Livers were then frozen immediately in liquid nitrogen and stored at −80°C for further analysis.

### Analysis of serum parameters

Fasting blood samples were collected prior to castration and twice weekly throughout the study. Sera were separated from collected blood samples by centrifugation at 3000 × *g* at 4°C for 15 min and stored at −80°C for further analysis. Serum testosterone concentrations were measured at week 7 (0 w; the start of the experimental period after the 7-week acclimation) and week 19 (12 w; the end of the experimental period), using a commercial radioimmunoassay kit (North Institute of Biological Technology, Beijing, China).

Serum samples were analyzed to determine serum lipid levels. Total cholesterol (TC), high-density lipoprotein cholesterol (HDL-C), low-density lipoprotein cholesterol (LDL-C), and total triglyceride (TG) were measured with commercial kits (Rongsheng Biotech, Co., Ltd., Shanghai, China) using an Automatic Biochemistry Analyzer (Hitachi 7020, Tokyo, Japan). Serum PCSK9 was measured using a commercial enzyme-linked immunoassay (ELISA) kit (HaiTai TongDa Sci Tech, Co., Ltd., Beijing, China) according to the manufacturer’s instructions.

### Biochemical analysis in liver tissues

Liver samples were studied to determine the hepatic TC and TG contents and enzyme activities. Hepatic lipids were measured as described by Shi et al. [21] with slight modifications. Briefly, liver samples from each pig were homogenized at 4°C in phosphate-buffered saline (pH 7.2). Liver samples were then centrifuged at 3000 × *g* for 10 min at 4°C, and TC and TG levels in the resulting supernatants were determined using commercially available kits (Rongsheng Biotech, Co., Ltd., Shanghai, China), according to the manufacturer’s instructions. Protein concentrations in liver samples were measured with a BCA Assay Kit (Pierce, Rockford, IL, USA).

Enzymatic HMGCR and CYP7A1 activities, as well as LDLR concentrations, present in the livers were measured using commercial ELISA kits (Biovol Biotech, Co., Ltd., Shanghai, China), as described previously [21].

### Quantitative real-time PCR analyses

Total RNA from liver specimens was isolated using TRIzol reagent (Invitrogen, Carlsbad, CA, USA) according to the manufacturer’s instructions. One microgram of total RNA was reverse transcribed into cDNA using an MMLV-RT Kit (Promega, Madison, WI, USA) according to the manufacturer’s protocol. Real-time PCR was performed using the StepOnePlus Real-Time PCR Detection System (Applied Biosystems, Inc., Foster City, CA, USA). The sequences of primers used for measuring mRNA expression levels are shown in Table 1. Amplifications were performed in 20-μL reaction mixtures containing 10.4 μL of 2× SYBR Premix Ex Taq (TaKaRa, Dalian, China), 0.4 μL of each primer (10 mM), 7.8 μL of distilled water, and 1.0 μL of cDNA. The following thermocycling conditions were used for all real-time PCR reactions: 95°C for 5 min, followed by 40 cycles of 95°C for 15 s, 60°C for 30 s, and 72°C for 30 s. After amplification, melt curve analysis was performed to confirm the reaction specificities. All measurements were performed in triplicate. β-Actin was used as a reference gene to normalize gene expression. The 2^-∆∆CT^ method was used to analyze the qRT-PCR data and assign relative expression differences [22].

**Table 1:** **Primers used for quantitative real-time RT-PCR**

**Gene**	**Primer sequences (5′-3′)**		**Gen bank accession no.**
LDLR	Forward	TCCAAGAGAAAGGCTCCAAG	NM_001206354.2
	Reversed	CTCCGTCACCAGCGAGTAG	
SREBP2	Forward	GATGGGCAGCAGAGTTCC	XM_005658510.1
	Reversed	ACAGCAGCAGGTCACAGGT	
LXRα	Forward	AGATGTCCTTGTGGGTGGAG	NM_001101814.1
	Reversed	CAGAGACTGGCTGCTTGC	
HMGCR	Forward	ACAGAAGCGATGGTTGAGGT	NM_001122988.1
	Reversed	AGATGGCAGTCACGATGTTG	
CYP7A1	Forward	GAATGACACCCTCTCCACCT	NM_001005352.2
	Reversed	GGAATAAGCACCAGAAAGTCG	
PCSK9	Forward	GAACCTGGAGCGGATTCTC	XM_003356453.2
	Reversed	CACTTTGGATGCTGGTGTCT	
β-Actin	Forward	ACTGGGACGACATGGAGAAGA	DQ178122
	Reversed	TTGGCTTTGGGGTTCAGG	

LDLR, low-density lipoprotein receptor; SREBP2, sterol regulatory element binding protein 2; LXRα, liver X receptor alpha; HMGCR, 3-hydroxy-3-methylgutaryl coenzyme A reductase; CYP7A1, cholesterol 7α-hydroxylase; PCSK9, proprotein convertase subtilisin/kexin type 9; β-Actin, beta-actin.

### Statistical analysis

The results are presented as mean ± SEM. The data were analyzed by one-way analysis of variance (ANOVA), followed by Tukey’s test. Correlation analysis was performed using Pearson’s test. P values of less than 0.05 were considered statistically significant. All statistical analyses were performed by using SPSS software, version 13.0 (SPSS, Chicago, IL, USA).

## Results

### Body weight, liver weight, and serum testosterone levels

Body weight, liver weight, and serum testosterone levels in pigs after 12 weeks of being fed an HFC diet are shown in Table 2. Initial body weights were similar in pigs in the IM + HFC, CM + HFC, and CM + HFC + T groups. CM + HFC pigs fed an HFC diet gained less weight than did pigs in the other groups. With testosterone replacement, the body weights of CM + HFC + T pigs increased and were similar to the body weights of pigs in the IM + HFC group (Table 2). Liver weight indexes in CM + HFC pigs were higher than those in the IM + HFC and CM + HFC + T groups, but these differences were not significant (*P* > 0.05).

**Table 2:** **Body weight, liver weight, and serum testosterone levels in pigs fed a high-fat and high-cholesterol diet** ^**a**^

	**IM + HFC**	**CM + HFC**	**CM + HFC + T**
Body weight (kg)			
Initial^**b**^	12.76 ± 0.45	12.14 ± 0.35	12.45 ± 0.89
Final^c^	25.15 ± 0.86	23.69 ± 0.64	26.22 ± 1.20
Serum T concentrations (ng/mL)			
Initial	7.82 ± 0.49	8.22 ± 0.56	8.78 ± 0.24
Final	3.52 ± 0.24^**^	0.25 ± 0.01	4.43 ± 0.31^##^
Liver weight (g)	483.82 ± 47.04	524.72 ± 69.88	558.38 ± 27.95
Liver weight index (%)	1.92 ± 0.17	2.20 ± 0.25	2.12 ± 0.39

Note: ^a^Data are presented as mean ± SEM; n = 6 pigs per group. **P <0.01 and ^##^ *P <0.01*, significant differences compared to the CM + HFC group determined by ANOVA (Tukey’s test). ^**b**^Initial: week 7 (0 w; the start of the experimental period after the 7-week acclimation). ^c^Final: week 19 (12 w; the end of the experimental period). T: testosterone.

Serum testosterone levels were not significantly different between groups before the study began. As expected, castration caused a significant decrease in serum testosterone levels. The concentration of serum testosterone at the end of the study was significantly lower in CM + HFC pigs than in IM + HFC pigs (*P* < 0.01). Testosterone treatment increased serum testosterone levels in CM + HFC + T pigs (*P* < 0.01; Table 2).

### Serum lipid profiles

**Figure 2:**
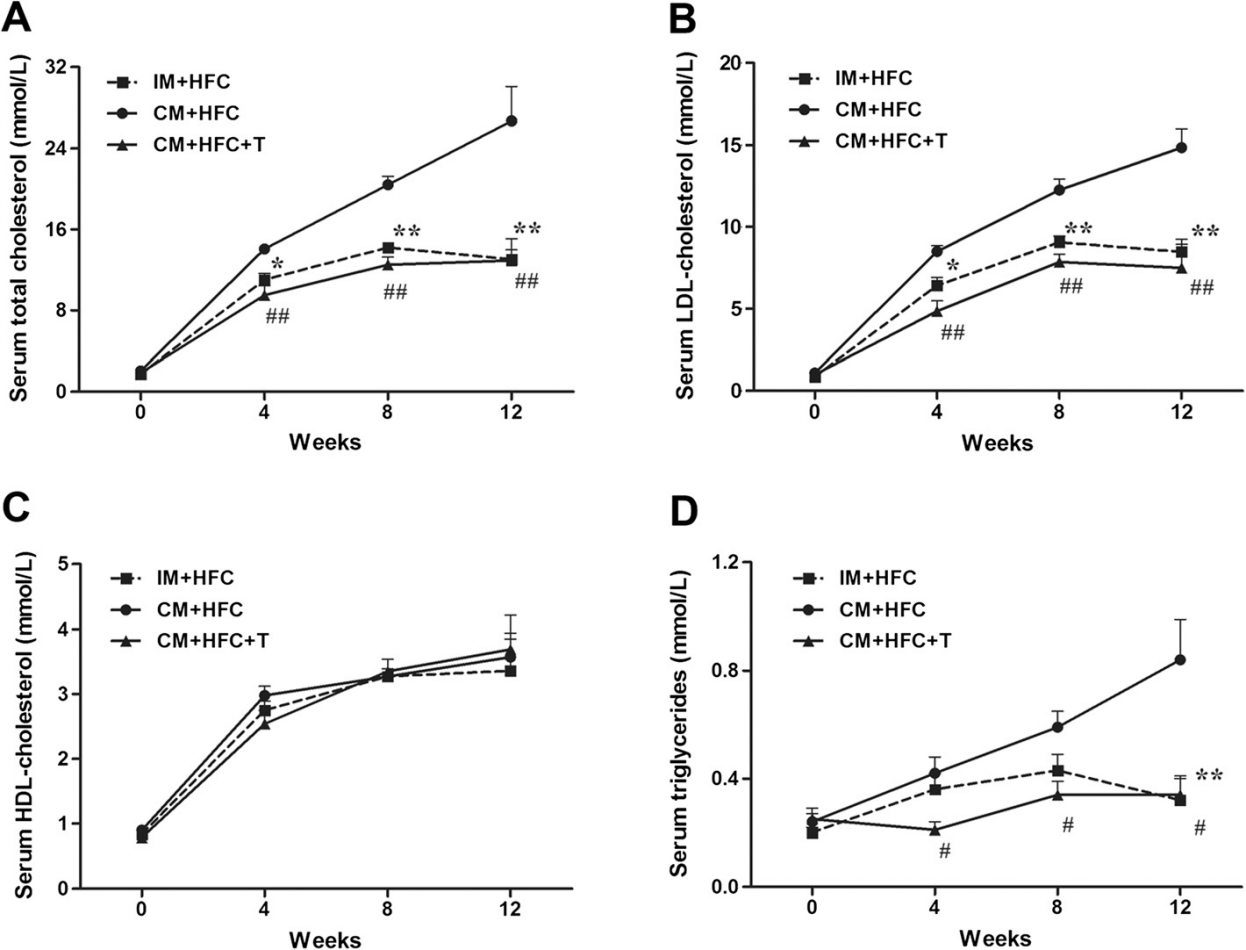
**Effects of castration and testosterone replacement on serum total cholesterol (A), LDL-cholesterol (B), HDL-cholesterol (C), and triglyceride (D) levels in pigs fed a high-fat and high-cholesterol diet.** Data are presented as mean ± SEM; n = 6 pigs per group. *P < 0.05 and **P < 0.01, IM + HFC vs. CM + HFC group; ^#^ *P* < 0.05 and ^##^ *P* < 0.01, CM + HFC + T vs. CM + HFC group. Significant differences were determined by analysis of variance (ANOVA; Tukey’s test).

To analyze the effects of castration and testosterone treatment on serum lipid profiles in HFC-fed pigs, serum lipids were measured at 0, 4, 8, and 12 weeks post castration (Figure 2). CM + HFC pigs had higher TC levels than did IM + HFC and CM + HFC + T pigs by the fourth week of HFC supplementation (*P* < 0.01; Figure 2A). There were no significant differences in serum TC levels between IM + HFC and CM + HFC + T pigs (*P* > 0.05). Serum LDL-C followed a pattern similar to that of TC in all groups of pigs (Figure 2B). However, testosterone deficiency caused by castration had no significant influence on serum HDL-C levels in HFC-fed pigs (Figure 2C). Serum TG levels in CM + HFC pigs were higher than those in IM + HFC pigs at week 12 (*P* < 0.01) and those in CM + HFC + T pigs at weeks 4, 8, and 12 (*P* < 0.05; Figure 2D).

### Hepatic lipid contents

**Figure 3:**
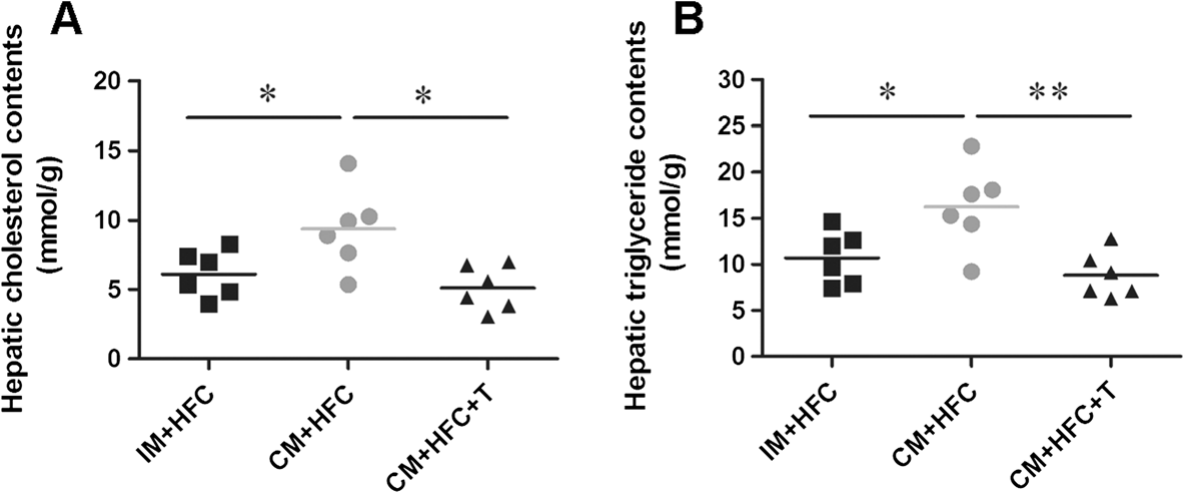
**Effects of castration and testosterone replacement on hepatic lipid contents in pigs fed a high-fat and high-cholesterol diet. (A)** Hepatic cholesterol contents. **(B)** Hepatic triglyceride contents. Data are presented as mean ± SEM; n = 6 pigs per group. *P < 0.05, **P < 0.01. Significant differences compared to the CM + HFC group determined by ANOVA (Tukey’s test).

As illustrated in Figure 3, hepatic cholesterol and triglyceride contents were also significantly higher in CM + HFC pigs than in IM + HFC pigs (Figure 3). Moreover, testosterone treatment attenuated the increase in hepatic lipid contents in CM + HFC pigs (Figure 3).

### Hepatic mRNA expressions of genes involved in cholesterol metabolism

**Figure 4:**
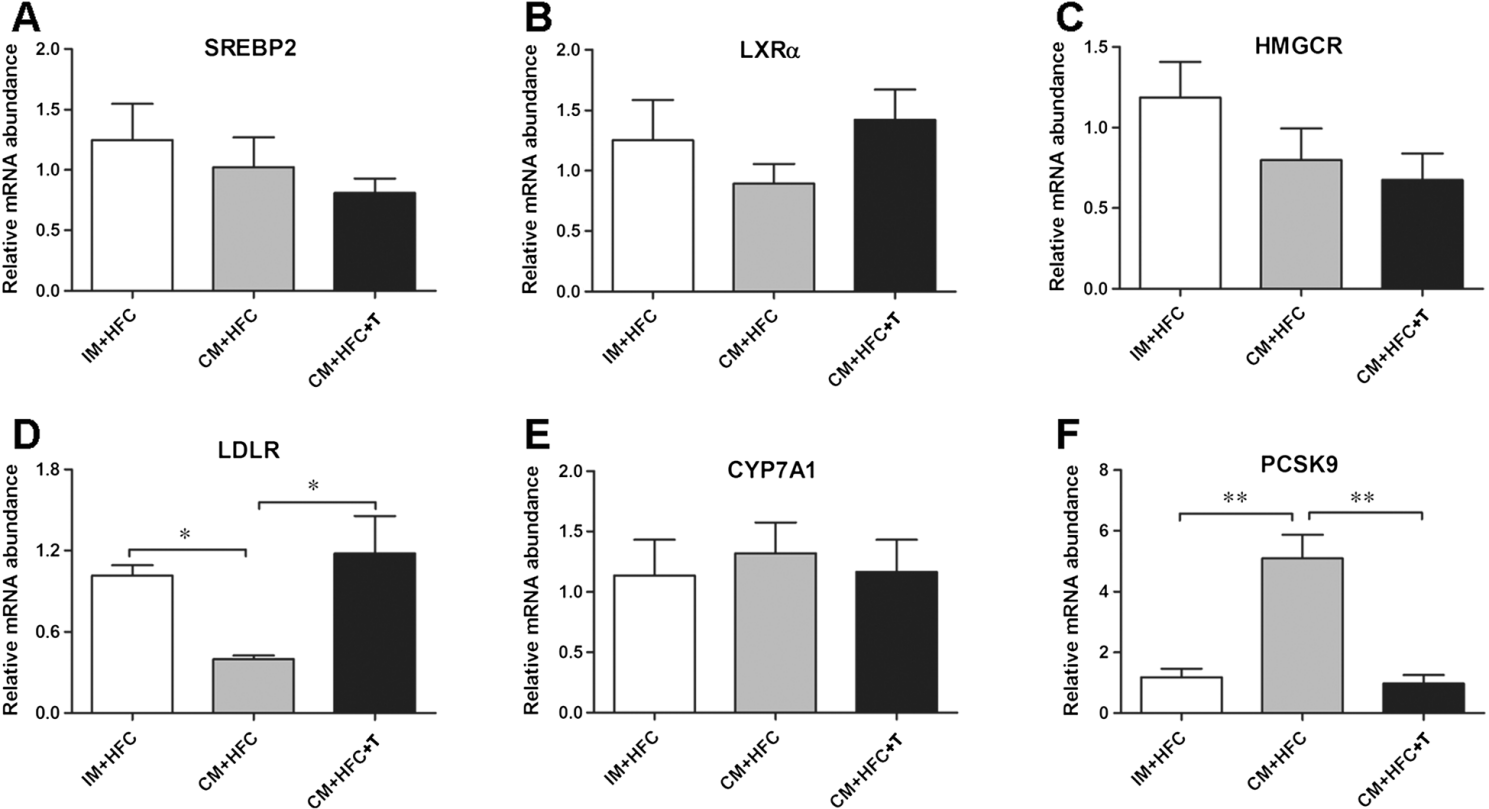
**Effects of castration and testosterone replacement on liver** ***SREBP2*** **(A),** ***LXRα*** **(B),** ***HMGCR*** **(C),** ***LDLR*** **(D),** ***CYP7A1*** **(E), and** ***PCSK9*** **(F) mRNA expression in pigs fed a high-fat and high-cholesterol diet.** SREBP2, sterol regulatory element binding protein 2; LXRα, liver X receptor alpha; HMGCR, 3-hydroxy-3-methylgutaryl coenzyme A reductase; LDLR, low-density lipoprotein receptor; CYP7A1, cholesterol 7α-hydroxylase; PCSK9, proprotein convertase subtilisin/kexin type 9. Data are presented as mean ± SEM; n = 6 pigs per group. *P < 0.05, significant differences compared to the CM + HFC group determined by ANOVA (Tukey’s test).

To investigate the effects of testosterone on the expression of genes controlling hepatic cholesterol metabolism, mRNA expression levels of *SREBP2*, *LXRα*, *HMGCR*, *LDLR*, *CYP7A1*, and *PCSK9* were studied by quantitative real-time PCR (Figure 4). The results showed that castration and testosterone replacement did not affect hepatic expression of *SREBP2*, *LXRα*, *HMGCR*, or *CYP7A1* at the mRNA level (Figure 4). However, castration markedly suppressed hepatic *LDLR* expression in CM + HFC pigs with respect to IM + HFC pigs and CM + HFC + T pigs (*P* < 0.05; Figure 4). In contrast, castration-induced testosterone deficiency induced a 4.3-fold increase in hepatic expression of *PCSK9* mRNA. Moreover, testosterone treatment restored increased expression of *PCSK9* in CM + HFC pigs (*P* < 0.01; Figure 4).

### Enzyme activities of HMGCR, LDLR, CYP7A1, and PCSK9

**Figure 5:**
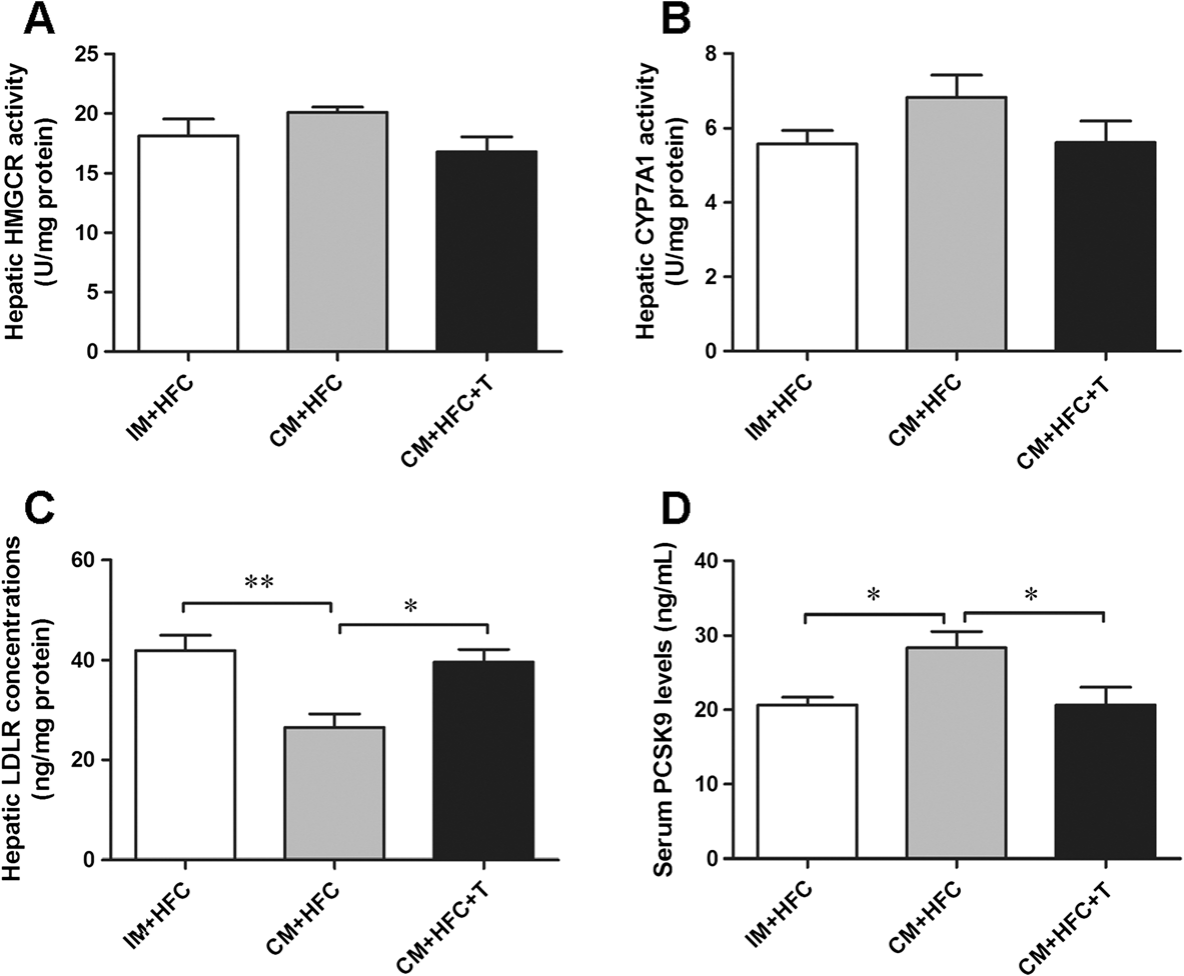
**Effects of castration and testosterone replacement on hepatic HMGCR (A), CYP7A1 (B) activities, LDLR concentrations (C), and serum PCSK9 levels (D) in pigs fed a high-fat and high-cholesterol diet.** HMGCR, 3-hydroxy-3-methylgutaryl coenzyme A reductase; CYP7A1, cholesterol 7α-hydroxylase; PCSK9, proprotein convertase subtilisin/kexin type 9. Data are presented as mean ± SEM; n = 6 pigs per group. *P < 0.05, **P < 0.01. Significant differences compared to the CM + HFC group determined by ANOVA (Tukey’s test).

Hepatic HMGCR and CYP7A1 activities, LDLR concentrations, and serum PCSK9 levels were measured, as shown in Figure 5. Evidently, neither castration nor testosterone treatment affected the activities of HMGCR or CYP7A1 in the liver of pigs (*P* > 0.05) (Figure 5A and 5B). However, testosterone deficiency significantly reduced hepatic LDLR protein levels in CM + HFC pigs (*P* < 0.01). Moreover, testosterone replacement reversed the reduction in hepatic LDLR levels in CM + HFC pigs (*P* < 0.05; Figure 5C). Serum PCSK9 levels were significantly increased by castration in pigs (*P* < 0.05), but this effect was reversed by testosterone replacement (Figure 5D).

### Correlations between serum PCSK9 levels and LDL-C and TC levels

**Figure 6:**
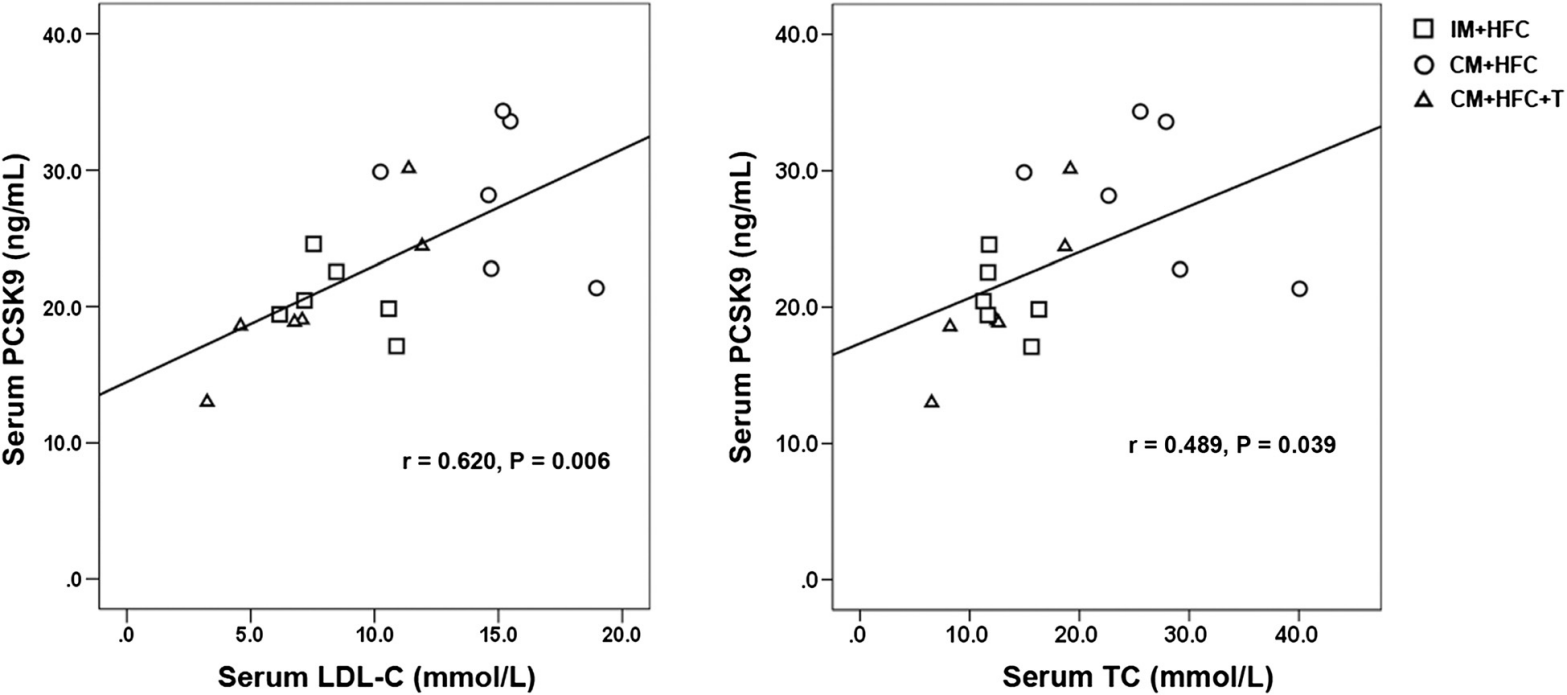
**Correlations between serum levels of PCSK9 and LDL-C or TC.** PCSK9, proprotein convertase subtilisin/kexin type 9; LDL-C, low-density lipoprotein cholesterol; TC, total cholesterol.

We next investigated the correlation between serum PCSK9 levels and LDL-C and TC levels in pigs fed an HFC diet. As shown in Figure 6, serum levels of PCSK9 were positively correlated with LDL-C (r = 0.620, *P* = 0.006) and TC (r = 0.489, *P* = 0.023).

## Discussion

In this study, we investigated the effects of testosterone levels on cholesterol metabolism in pigs fed an HFC diet. Our data indicated that CM + HFC pigs gained less weight than did pigs in the other groups. Testosterone deficiency was associated with a loss of lean muscle mass [20,23], which constitutes approximately 50% of the body mass in pigs [24]. The reduced weight of CM + HFC pigs may be attributable to the loss of skeletal muscle mass induced by castration. Serum cholesterol (total cholesterol and LDL-cholesterol) levels were significantly higher in CM + HFC pigs than in IM + HFC pigs. However, testosterone replacement attenuated these effects in CM + HFC pigs, suggesting that testosterone may play a protective role in diet-induced hypercholesterolemia. To the best of our knowledge, the present study is the first to demonstrate that testosterone deficiency induced by castration aggravates diet-induced hypercholesterolemia in a porcine model. In previous studies, researchers have used rodent models to evaluate the effects of testosterone on serum cholesterol levels; however, the observed relationships between testosterone and serum cholesterol levels are inconclusive [13,25,26]. In addition, the process of lipoprotein metabolism differs greatly between rodents and humans [27]. Pigs may be more relevant to the study of cholesterol metabolism because of their high degree of similarity with humans in terms of lipid metabolism and cardiovascular physiology [27-29], making them an attractive alternative to rodents. Importantly, our previous study revealed that castration-induced testosterone deficiency results in elevated serum cholesterol levels in pigs fed a normal diet [20], suggesting that pigs are suitable models for studying the effects of sex hormones on cholesterol metabolism.

In addition to its effects on serum cholesterol levels, testosterone deficiency also caused a significant increase in serum TG levels in CM + HFC pigs. TGs in the liver are packaged into very low-density lipoproteins (VLDLs) and exported to the circulation. Excessive production of TG-rich VLDL (VLDL-TG) contributes to hypertriglyceridemia [30]. Many genes are involved in the assembly and secretion of VLDL-TG, such as the microsomal triglyceride transfer protein (MTP) and apolipoprotein A-V (apoA-V) [31,32]. Previous studies showed that testosterone regulates hepatic MTP expression in rats and mice [25,33]. ApoA-V was recently identified as an important modulator of TG metabolism and shown to lower plasma TG levels in both humans and animals [31,34]. The results of recent studies have shown decreased apoA-V expression in obese subjects. Moreover, the treatment of HepG2 cells with insulin decreased apoA-V expression [34,35]. Testosterone deficiency is associated with obesity and insulin resistance [36,37]. Thus, testosterone deficiency may affect serum TG levels by altering the expression of genes involved in lipoprotein assembly and secretion. The role of testosterone in regulating TG levels was not the primary goal of our study; although this topic will be investigated in future studies.

Little is known regarding the molecular mechanisms underlying the effects of testosterone deficiency on cholesterol metabolism. Cholesterol homeostasis is controlled by coordinated changes in the expression of multiple genes involved in cholesterol biosynthesis, uptake, and efflux [21]. It was well known that LXRα plays a critical role in cholesterol metabolism [7] and that *LXRα* expression increases cholesterol efflux by regulating its target genes. CYP7A1 is one of the most important target genes of LXRα and was identified as the rate-limiting enzyme in bile acid-synthesis for cholesterol elimination [15]. In the present study, we observed that castration had no effect on the liver mRNA expression levels of *LXRα* or *CYP7A1* in pigs. These results suggested that the increased cholesterol levels observed in CM + HFC pigs may not have resulted from enhanced cholesterol efflux through the LXRα-CYP7A1 pathway. HMGCR is the rate-limiting enzyme mediating de novo cholesterol synthesis [38]. Similarly, hepatic HMGCR activity and mRNA expression were not changed by castration and testosterone replacement. Thus, testosterone deficiency might not influence the de novo synthesis of cholesterol in the livers of CM + HFC pigs. It is well known that LDLR is a crucially important modulator of plasma LDL-cholesterol levels in humans and animals. Increased LDLR expression was shown to result in reduced serum LDL-cholesterol levels [21]. We found that LDLR mRNA expression and protein levels were significantly decreased in the livers of CM + HFC pigs, compared to IM + HFC and CM + HFC + T pigs. This agreed with our findings that CM + HFC pigs showed a significant increase in both serum LDL-cholesterol and TC levels. Moreover, these findings suggest that one of the mechanisms through which testosterone deficiency can increase serum cholesterol levels in CM + HFC pigs is by decreasing LDLR expression, thus inhibiting the removal of LDL-cholesterol from the circulation. In animals, hepatic *LDLR* and *HMGCR* are transcriptionally regulated by *SREBP2* [15]. However, in the present study hepatic *SREBP2* mRNA expression was not influenced by castration and testosterone replacement, suggesting that other mechanisms may participate in the regulation of *LDLR*. Previous studies demonstrated that hepatic *LDLR* mRNA transcription can be suppressed by a high-cholesterol diet [39-41]. Moreover, dietary cholesterol has been reported to increase hepatic TG and cholesterol stores, which in turn suppresses hepatic *LDLR* mRNA levels [42]. Thus, the reduced hepatic *LDLR* mRNA expression observed in CM + HFC pigs may have resulted from an increase in hepatic lipid stores induced by testosterone deficiency and an HFC diet. Recent studies have demonstrated a central role of PCSK9 in the regulation of cholesterol metabolism by enhancing the degradation of hepatic LDLR, resulting in increased serum LDL-cholesterol concentrations [43,44]. Moreover, results from previous studies indicated that the hormonal and dietary regulation of hepatic LDLR involves posttranscriptional regulation by PCSK9 [45,46]. Of note, we observed that *PCSK9* mRNA expression in liver was significantly increased in CM + HFC pigs compared to IM + HFC and CM + HFC + T pigs. This observation suggested that reduced hepatic LDLR protein levels following castration in CM + HFC pigs may occur via increased PCSK9 expression. In agreement with our findings, data from a recent study demonstrated a reduction in hepatic LDLR levels and severe hypercholesterolemia in *PCSK9*-transgenic pigs [29]. Taken together, our findings indicate that testosterone deficiency can increase serum LDL-cholesterol and total cholesterol levels by altering the expression of PCSK9 and LDLR, and might increase the risk of atherosclerosis and cardiovascular disease.

This study has several limitations. Only a small set of pigs were included in the study owing to practical issues associated with obtaining the animals. Moreover, we did not measure hepatic testosterone (T) and 17β-estradiol (E2) concentrations. Although testosterone deficiency can increase serum cholesterol levels in HFC-fed pigs, it should be considered that testosterone may be partially effective after being aromatized to estrogen [25]. The direct effects of testosterone were not examined in this study. In addition, we only examined the expression patterns of several key genes involved in cholesterol metabolism. Studying a select few genes cannot rigorously define the genomic mechanisms through which testosterone deficiency induces increased serum cholesterol levels. High-throughput technologies might be needed to assess castration-induced hepatic transcriptome changes in HFC-fed pigs. These issues should be addressed in future studies.

## Conclusions

In conclusion, the present study demonstrated that castration-induced testosterone deficiency caused a significant increase in serum cholesterol levels in pigs fed an HFC diet, and these effects were reversed by testosterone replacement. Importantly, testosterone deficiency could induce PCSK9 expression and reduce LDLR expression in the livers of CM + HFC pigs. The findings suggest that the increased serum cholesterol levels observed in CM + HFC pigs may be attributed in part to impaired LDL-cholesterol clearance. The precise mechanisms regulating the hypercholesterolemic effects of testosterone deficiency await more detailed investigation.

## Abbreviations

ANOVA: Analysis of variance
CM + HFC: Castrated male pigs fed a high-fat and high-cholesterol diet
CM + HFC + T: Castrated male pigs with testosterone replacement fed a high-fat and high-cholesterol diet
CYP7A1: Cholesterol 7α-hydroxylase
HDL-C: High-density lipoprotein cholesterol
HFC: High-fat and high-cholesterol
HMGCR: 3-hydroxy-3-methylgutaryl coenzyme A reductase
IM + HFC: Intact male pigs fed a high-fat and high-cholesterol diet
LDL-C: Low-density lipoprotein cholesterol
LDLR: Low-density lipoprotein receptor
LXRα: Liver X receptor alpha
PCSK9: Proprotein convertase subtilisin/kexin type 9
SREBP2: Sterol regulatory element binding protein 2
TC: Total cholesterol
TG: Triglyceride

## References

[1] Feldman HA, Longcope C, Derby CA, Johannes CB, Araujo AB, Coviello AD (2002). Age trends in the level of serum testosterone and other hormones in middle aged men: longitudinal results from the Massachusetts male aging study. J Clin Endocrinol Metab.

[2] Jones TH (2010). Testosterone deficiency: a risk factor for cardiovascular disease?. Trends Endocrinol Metab.

[3] Hak AE, Witteman JC, de Jong FH, Geerlings MI, Hofman A, Pols HA (2002). Low levels of endogenous androgens increase the risk of atherosclerosis in elderly men: the Rotterdam study. J Clin Endocrinol Metab.

[4] Traish AM, Kypreos KE (2011). Testosterone and cardiovascular disease: an old idea with modern clinical implications. Atherosclerosis.

[5] Ruige JB, Ouwens DM, Kaufman JM (2013). Beneficial and adverse effects of testosterone on the cardiovascular system in men. J Clin Endocrinol Metab.

[6] Huang XQ, Tang J, Zhou Q, Lu HP, Wu YL, Wu WK (2010). Polysaccharide from Fuzi (FPS) prevents hypercholesterolemia in rats. Lipids Health Dis.

[7] He WS, Wang MG, Pan XX, Li JJ, Jia CS, Zhang XM (2013). Role of plant stanol derivatives in the modulating of cholesterol metabolism and liver gene expression in mice. Food Chem.

[8] Traish AM, Abdou R, Kypreos KE (2009). Androgen deficiency and atherosclerosis: The lipid link. Vascul Pharmacol.

[9] Isidori AM, Giannetta E, Greco EA, Gianfrilli D, Bonifacio V, Isidori A (2005). Effects of testosterone on body composition, bone metabolism and serum lipid profile in middle-aged men: a meta-analysis. Clin Endocrinol (Oxf).

[10] Mäkinen JI, Perheentupa A, Irjala K, Pöllänen P, Mäkinen J, Huhtaniemi I (2008). Endogenous testosterone and serum lipids in middle-aged men. Atherosclerosis.

[11] Zhang N, Zhang H, Zhang X, Zhang B, Wang F, Wang C (2014). The relationship between endogenous testosterone and lipid profile in middle-aged and elderly Chinese men. Eur J Endocrinol.

[12] Lee CE, Kang JS, Kim KI (2008). Effects of gender, gonadectomy and sex hormones on growth and plasma cholesterol level in rats. Ann Nutr Metab.

[13] Hatch NW, Srodulski SJ, Chan HW, Zhang X, Tannock LR, King VL (2012). Endogenous androgen deficiency enhances diet-induced hypercholesterolemia and atherosclerosis in low-density lipoprotein receptor-deficient mice. Gend Med.

[14] Yang L, Chen JH, Xu T, Nie MH, Yang HK (2013). Hypocholesterolemic effect of rice protein is due to regulating hepatic cholesterol metabolism in adult rats. Gene.

[15] Liang YT, Wong WT, Guan L, Tian XY, Ma KY, Huang Y (2011). Effect of phytosterols and their oxidation products on lipoprotein profiles and vascular function in hamster fed a high cholesterol diet. Atherosclerosis.

[16] van Eerdenburg FJ, Lugard-Kok CM, Dieleman SJ, Bevers MM, Swaab DF (1991). Influence of gonadectomy and testosterone supplementation on the postnatal development of the vasopressin and oxytocin-containing nucleus of the pig hypothalamus. Neuroendocrinology.

[17] Kojima M, Sekimoto M, Degawa M (2008). A novel gender-related difference in the constitutive expression of hepatic cytochroma P4501A subfamily enzymes in Meishan pigs. Biochem Pharmacol.

[18] Khan MA, Earl FL, Farber TM, Miller E, Husain MM, Nelson E (1977). Elevation of serum cholesterol and increased fatty streaking in egg yolk:lard fed castrated miniature pigs. Exp Mol Pathol.

[19] Jacobsson L (1986). Comparison of experimental hypercholesterolemia and atherosclerosis in Gottingen mini-pigs and Swedish domestic swine. Atherosclerosis.

[20] Yao YC, Cai ZW, Zhao CJ, Wu KL, Wu CX, Han WP (2011). Influence of castration-induced sex hormone deficiency on serum lipid levels and the genes expression in male pigs. Horm Metab Res.

[21] Shi Y, Guo R, Wang X, Yuan D, Zhang S, Wang J (2014). The regulation of alfalfa saponin extract on key genes involved in hepatic cholesterol metabolism in hyperlipidemic rats. PLoS One.

[22] Livak KJ, Schmittgen TD (2001). Analysis of relative gene expression data using real-time quantitative PCR and the 2(−Delta Delta C(T)) Method. Methods.

[23] Christoffersen B, Raun K, Svendsen O, Fledelius C, Golozoubova V (2006). Evaluation of the castrated male Sprague–Dawley rat as a model of the metabolic syndrome and type 2 diabetes. Int J Obes (Lond).

[24] Liaubet L, Lobjois V, Faraut T, Tircazes A, Benne F, Iannuccelli N (2011). Genetic variability of transcript abundance in pig peri-mortem skeletal muscle: eQTL localized genes involved in stress response, cell death, muscle disorders and metabolism. BMC Genomics.

[25] Senmaru T, Fukui M, Okada H, Mineoka Y, Yamazaki M, Tsujikawa M (2013). Testosterone deficiency induces markedly decreased serum triglycerides, increased small dense LDL, and hepatic steatosis mediated by dysregulation of lipid assembly and secretion in mice fed a high-fat diet. Metabolism.

[26] Constantinou C, Mpatsoulis D, Natsos A, Petropoulou PI, Zvintzou E, Traish AM (2014). The low density lipoprotein receptor modulates the effects of hypogonadism on diet-induced obesity and related metabolic perturbations. J Lipid Res.

[27] Wei J, Ouyang H, Wang Y, Pang D, Cong NX, Wang T (2012). Characterization of a hypertriglyceridemic transgenic miniature pigs model expressing human apolipoprotein CIII. FEBS J.

[28] Rapacz J, Hasler-Rapacz J, Taylor KM, Checovich WJ, Attie AD (1986). Lipoprotein mutations in pigs are associated with elevated plasma cholesterol and atherosclerosis. Science.

[29] AI-Mashhadi RH, Sørensen CB, Kragh PM, Christoffersen C, Mortensen MB, Tolbod LP (2013). Fimilial hypercholesterolemia and atherosclerosis in cloned minipigs created by DNA transposition of human PCSK9 gain-of-function mutant. Sci Transl Med.

[30] Kim DH, Zhang T, Lee S, Calabuig-Navarro V, Yamauchi J, Piccirillo A (2014). FoxO6 integrates insulin signaling with MTP for regulating VLDL production in the liver. Endocrinology.

[31] Blade AM, Fabritius MA, Hou L, Weinberg RB, Shelness GS (2011). Biogenesis of apolipoprotein A-V and its impact on VLDL triglyceride secretion. J Lipid Res.

[32] Han CC, Wang JW, Pan ZX, Tang H, Xiang SX, Wang J (2013). Effect of cholesterol on lipogenesis and VLDL-TG assembly and secretion in goose primary hepatocytes. Mol Cell Biochem.

[33] Améen C, Oscarsson J (2003). Sex difference in hepatic microsomal triglyceride transfer protein expression is determined by the growth hormone secretory pattern in rat. Endocrinology.

[34] Nilsson SK, Heeren J, Olivecrona G, Merkel M (2011). Apolipoprotein A-V; a potent triglyceride reducer. Atherosclerosis.

[35] Huang XS, Zhao SP, Hu M, Bai L, Zhang Q, Zhao W (2010). Decreased apolipoprotein A5 is implicated in insulin resistance-related hypertrilyceridemia in obesity. Atherosclerosis.

[36] Rao PM, Kelly DM, Jones TH (2013). Testosterone and insulin resistance in the metabolic syndrome and T2DM in men. Nat Rev Endocrinol.

[37] Christoffersen BO, Gade LP, Golozoubova V, Svendsen O, Raun K (2010). Influence of castration-induced testosterone and estrodiol deficiency on obesity and glucose metabolism in male Göttigen minipigs. Steroids.

[38] Bose-Boyd RA (2008). Feedback regulation of cholesterol synthesis: sterol-accelerated ubiquitination and degradation of HMG CoA reducase. Cell Res.

[39] Srivastava RA, Jiao S, Tang JJ, Pfleger BA, Kitchens RT, Schonfeld G (1991). In vivo regulation of low-density lipoprotein receptor and apoliprotein B gene expressions by dietary fat and cholesterol in inbred strains of mice. Biochim Biophys Acta.

[40] Huang Z, Zhou X, Nicholson AC, Gotto AM, Hajjar DP, Han J (2008). Activation of peroxisome proliferator-activated receptor-alpha in mice induces expression of the hepatic low-density lipoprotein receptor. Br J Pharmacol.

[41] Kawaguchi H, Yamada T, Miura N, Ayaori M, Uto-Kondo H, Ikegawa M (2014). Rapid development of atherosclerosis in the world’s smallest Microminipig fed a high-fat/high-cholesterol diet. J Atheroscler Thromb.

[42] Hennessy LK, Osada J, Ordovas JM, Nicolosi RJ, Stucchi AF, Brousseau ME (1992). Effects of dietary fats and cholesterol on liver lipid content and hepatic apolipoprotein A-I, B, and E and LDL receptor mRNA levels in cebus monkeys. J Lipid Res.

[43] Miranda MX, van Tits LJ, Lohmann C, Arsiwala T, Winnik S, Tailleux A, et al: The Sirt1 activator SRT3025 provides atheroprotection in Apoe−/− mice by reducing hepatic Pcsk9 secretion and enhancing Ldlr expression. Eur Heart J. in press. 10.1093/eurheartj/ehu095 PMC4286317 24603306

[44] Tai MH, Chen PK, Chen PY, Wu MJ, Ho CT, Yen JH (2014). Curcumin enhances cell-surface LDLR level and promotes LDL uptake through downregulation of PCSK9 gene expression in HepG2 cells. Mol Nutr Food Res.

[45] Persson L, Gälman C, Angelin B, Rudling M (2009). Importance of proprotein convertase subtilisin/kexin type 9 in the hormonal and dietary regulation of rat liver low density lipoprotein receptors. Endocrinology.

[46] Persson L, Henriksson P, Westerlund E, Hovatta O, Angelin B, Rudling M (2012). Endogenous estrogens lower plasma PCSK9 and LDL cholesterol but not Lp(a) or bile acid synthesis in women. Arterioscler Thromb Vasc Biol.

